# The Effect of Early Life Exposure to Triclosan on Thyroid Follicles and Hormone Levels in Zebrafish

**DOI:** 10.3389/fendo.2022.850231

**Published:** 2022-06-03

**Authors:** Ning Tang, Pianpian Fan, Li Chen, Xiaogang Yu, Wenjuan Wang, Weiye Wang, Fengxiu Ouyang

**Affiliations:** ^1^ Ministry of Education and Shanghai Key Laboratory of Children’s Environmental Health, Xinhua Hospital, Shanghai Jiao Tong University School of Medicine, Shanghai, China; ^2^ Guangxi Key Laboratory of Tumor Immunology and Microenvironmental Regulation, Guilin Medical University, Guilin, China

**Keywords:** triclosan, early exposure, thyroid hormone, thyroid follicles, zebrafish

## Abstract

Triclosan (TCS) is an antimicrobial chemical widely used in personal care products. Most of the TCS component is discharged and enters the aquatic ecosystem after usage. TCS has a similar structure as thyroid hormones that are synthesized by thyroid follicular epithelial cells, thus TCS has a potential endocrine disrupting effect. It is still not clear how the different levels of the environmental TCS would affect early development *in vivo*. This study examines the effects of TCS on thyroid hormone secretion and the early development of zebrafish. The fertilized zebrafish eggs were exposed to TCS at 0 (control), 3, 30, 100, 300, and 900 ng/mL, and the hatching rate and the larvae mortality were inspected within the first 14 days. The total triiodothyronine (TT_3_), total thyroxine (TT_4_), free triiodothyronine (FT_3_), and free thyroxine (FT_4_) were measured at 7, 14, and 120 days post-fertilization (dpf). The histopathological examinations of thyroid follicles were conducted at 120 dpf. TCS exposure at 30-300 ng/mL reduced the hatching rate of larvae to 34.5% to 28.2 % in the first 48 hours and 93.8 .7 % to 86.8 % at 72 h. Extremely high TCS exposure (900 ng/mL) strongly inhibited the hatching rate, and all the larvae died within 1 day. Exposure to TCS from 3 to 300 ng/mL reduced the thyroid hormones production. The mean TT_3_ and FT_3_ levels of zebrafish decreased in 300 ng/mL TCS at 14 dpf (300 ng/mL TCS vs. control : TT_3_ , 0.19 ± 0.08 vs. 0.39 ± 0.06; FT_3_, 19.21 ± 3.13 vs. 28.53 ± 1.98 pg/mg), and the FT_4_ decreased at 120 dpf ( 0.09 ± 0.04 vs. 0.20 ± 0.14 pg/mg). At 120 dpf , in the 300 ng/mL TCS exposure group, the nuclear area and the height of thyroid follicular epithelial cells became greater, and the follicle cell layer got thicker. This happened along with follicle hyperplasia, nuclear hypertrophy, and angiogenesis in the thyroid. Our study demonstrated that early life exposure to high TCS levels reduces the rate and speed of embryos hatching, and induces the histopathological change of thyroid follicle, and decreases the TT_3_, FT_3_, and FT_4_ production in zebrafish.

## Introduction

Thyroid hormones are synthesized and secreted by the thyroid follicular epithelial cells, and are critical for fetal growth and neuro-development in early life (1, 2). The thyroid gland is sensitive to environmental endocrine disruptor chemicals (EDCs) during early life (3). Triclosan (TCS) is a widely used antimicrobial agent in personal care products with a similar molecular structure as the thyroid hormone, thus it is a potential EDC. TCS has been widely used in personal care products, such as soaps, toothpastes, shampoos, and cosmetics for more than 40 years (4, 5). Most of TCS is discharged into residential drains after usage, and contaminates the aquatic ecosystem (6, 7). TCS can be detected in rivers, lakes (8), and drinking water (9). The highest TCS concentrations can reach up to 5370 ng/L in effluent water of wastewater treatment plants (10) and 1023 ng/L in rivers (11). TCS is bio-accumulated in fish and other aquatic species (12–14), and can also be detected in human urine, blood, fetal cord blood, and breast milk (15–17), suggesting there is a wide exposure to TCS of the general population in different regions of the world. For example, TCS has been detected in pregnant women with the median urinary concentration ranging from 0.4 μg/L to 26.5 μg/L (18–22).

Concerns have been raised on the potential risk for TCS to perturb thyroid endocrine functioning, in part, due to the structural similarities of TCS and thyroid hormones (23). Several animal studies had examined the potential thyroid-disrupting effect of TCS in rodents (24, 25). Most of the rodent studies found that TCS exposure decreased the serum total thyroxine (T_4_) and triiodothyronine (T_3_) levels (26, 27) at the doses ranging from 1 to 300 mg/kg/d. Considering TCS is a persistent and ubiquitous pollutant in the aquatic environment, the thyroid disturbing effect of TCS at different aquatic relevant concentrations needs to be studied. But few studies have directly evaluated the effect of TCS early exposure in fish (28, 29). Moreover, the histopathologic examination is considered the gold standard for evaluating the pathological changes in tissues and organs (30), but few studies have explored the impact of TCS exposure on thyroid follicular pathological changes in fish.

Zebrafish has been a good model for studies of thyroid function, since the development of its thyroid system is comparable to human (31). None has examined the effect of early exposure to TCS from the embryo stage at the environment-related level or the bio-sample concentrations, on the thyroid gland and the related hormone levels. In this study, by using a zebrafish model, we examined the impacts of early exposure to TCS at environmentally relevant concentrations on the histopathology of thyroid follicles and the thyroid hormones levels.

## Materials and Methods

### TCS

TCS (Irgasan, 5-chloro-2-(2, 4-dichlorophenoxy) phenol, ≥ 97.0 % purity (HPLC), CAS no. 3380-34-5) and dimethyl sulfoxide (DMSO, 99 % purity) were purchased from Sigma-Aldrich (St. Louis, MO).

### Zebrafish Maintenance and TCS Exposure

Zebrafish strains of AB wild-type line were used in the study. The male and female zebrafish at 5-months-old were acclimated in tanks containing dechlorinated tap water for 4 weeks before mating under a photoperiod of 14:10 h light/dark cycle. The fish were fed with brine shrimp twice per day.

Fertilized eggs were collected within 30 min after natural mating and unfertilized eggs were discarded. Fertilized eggs were randomly assigned to 0 (0.01 % DMSO as solvent control), 3, 30, 100, 300, and 900 ng/mL TCS (600 eggs per group) and raised up to 7 and 14 days at 28.0 ± 0.5°C under 14:10 h light/dark photoperiod cycle (32). These TCS concentrations were set based on the environmental exposure levels of both humans and wildlife (17, 33). There was 0.01% DMSO used as a solvent to enhance TCS solubility (34), as the control group . The zebrafish embryos were tolerant to low concentrations (0.01 %) of DMSO (35).

There were 200 fertilized eggs with a density of one embryo per 2 mL medium put into a culture dish until 72 h post-fertilization (hpf). The larvae were transferred into glass tanks with a density of one larva per 60 mL medium until 14 days post-fertilization (dpf) (36). After 14 dpf, the zebrafish were transferred into larger glass tanks with a density of one fish per 200 mL medium (36). Half of the medium was changed twice per day to ensure TCS concentration stable. The number of larvae hatched were inspected and recorded twice per day in the first 3 dpf (i.e., 72 hpf). The larvae were fed with paramecium twice per day until 14 dpf. Starting from 15 dpf, larvae were fed brine shrimp. The pH of raising water was maintained at 7.5 ± 0.5, conductivity was maintained at 550 ± 50 μS, temperature was maintained at 28 ± 0.5°C , and the dissolved oxygen of the raising water was monitored (32).

### Hatching Rate and Mortality

The hatching rate was calculated as the number of larvae hatched during the first 3 days divided by the total number of fertilized eggs. The death of larvae was inspected and recorded twice per day for first 14 days, and the dead larvae were removed from dishes/tanks. The mortality was calculated as the number of dead larvae divided by the total number of hatched larvae.

### Thyroid Hormone Measurements

At 7, 14, and 120 dpf, the zebrafish were euthanized by immersing in ice-cold water for 40 min (37, 38), and thyroid hormones (THs) were measured by ELISA (enzyme-linked immunosorbent assay) kits. Specifically, at 7 dpf, 50 larvae were homogenated and put together for one measurement. Three replicates (50 larvae each replicate) were conducted. At 14 dpf, 100 larvae were gathered for each replicate for THs measurements. For the zebrafish at 120 dpf, the heads were removed for thyroid histological analysis, and the rest of fish body was used for thyroid hormones measurement.

The free triiodothyronine (FT_3_), free thyroxine (FT_4_), total triiodothyronine (TT_3_), and total thyroxine (TT_4_) levels were measured by ELISA (Labor Diagnostika Nord commercial kit, Nordhorn, Germany) (39). The larvae samples (at 7 and 14 dpf) and the body of the fish (with head, viscera, and gut removed, at 120 dpf) were sonicated in cold 0.01 M phosphate buffer saline (PBS) at w/v of 1mg/5μL (w: wet weight of samples, v: volume of 0.01 M PBS, mg/μL), put on intermittent sonic oscillation for 5 min, vortexed vigorously for 10 min, then centrifuged at 12,000 × g at 4°C for 5 min. The supernatant was collected to measure thyroid hormones and total protein concentration. Total protein concentration was measured with the Pierce^©^ BCA Protein Assay Kit (Thermo Fisher Scientific Inc., Rockford, IL) to normalize thyroid hormone concentration (40). The limit of detection (LOD) was 0.1 ng/mL for TT_3_, 8 nmol/L for TT_4_, 0.3 pg/mL for FT_3_, and 1 pg/mL for FT_4_. Values below the limit of detection were replaced with values equal to the LOD divided to 2.

### Hematoxylin and Eosin (H&E) Staining of Thyroid Gland and Histological Analysis

The histological analysis of the thyroid gland was conducted in zebrafish at 120 dpf. The heads of zebrafish were fixed in 10% neutral formalin (Zhongshan Beijing Biotechnology Co., Ltd., Beijing, China) for at least 12 h, and transferred to 70% ethanol, all at room temperature. Each zebrafish head was placed in processing cassettes, dehydrated through a serial alcohol gradient, and embedded in the wax blocks.

Serial transverse cross-sections were cut using a microtome 5 μm and dewaxed in xylene, rehydrated through decreasing concentrations of ethanol, and washed in PBS. The sections were then stained with hematoxylin for 4−5 min and with eosin for 1−2 min (Zhongshan Beijing Biotechnology Co., Ltd.). Thyroid follicles were located according to their being dispersed among the afferent branchial arterioles (for example, ventral aorta) in the subpharyngeal region (41). Stained thyroid follicle sections were photographed under light microscopy (BX53−p; Olympus Corporation, Tokyo, Japan).

### The Nuclear Size and Height of Follicular Epithelial Cells

Photographs of each follicle were taken at the largest follicle diameter (determined by observing serial sections) with an Olympus digital camera (BX53−p; Olympus Corporation). The height of follicular epithelial cells and the long and short diameters of their cell nuclei were quantitatively analyzed. The nuclear size was calculated as the long diameter × short diameter × π/4 (41). At least 3-5 histological tissue sections per one fish sample and three different follicles (or three separate areas) per one section were selected for measuring in cases where the follicular structure was unclear or absent. The nuclear size was calculated in at least 50 thyroid follicular cell nuclei per fish at a magnification of ×1000. The height of a follicular epithelial cell was calculated by the mean of five measures along the follicle perimeter at equal intervals, and 40-80 follicular epithelial cells were measured for each fish (42). Image Pro−Plus 6.0 software (Media Cybernetics, Inc., Rockville, MD) was used to analyze the photos.

### Statistical Analysis

The ANOVA F-test was used to compare the differences in thyroid hormone levels, the height of thyroid follicle epithelial cells and nuclear size, and chi square test was used to compare the difference in hatching rate and mortality of larvae among TCS exposure groups. We used linear regression models to evaluate the associations of TCS exposures with thyroid hormones and thyroid follicle histopathological changes. The level of significance was two-sided *P* value < 0.05. All analyses were performed using the SAS 9.3 software (SAS Institute, Inc., Cary, NC).

## Results

### TCS Exposure and Hatching Rate of Larva

All the hatched larvae presented a well-developed head, body, and tail within 72 h. At 48 hpf, the hatching rate was 42% in the control group, and 47.8 % in 3 ng/ml TCS exposure group . But with the increase of TCS exposure concentrations , the hatching rate significantly dropped from 34.5 % to 4.3 % in 30 - 900 ng/mL TCS exposure (*P* < 0.01. **Table 1**). At 72 hpf, the hatching rate was 95.8 % in the control group, while the hatching rates in 3-300 ng/mL TCS exposure groups slightly reduced as TCS dose increased, but the differences were not statistically significant compared to the control. However, 900 ng/mL of TCS exposure strongly inhibited the hatching rate: only 4.3 % of fertilized eggs hatched at 48 hpf, and 18.3 % at 72 hpf (**Table 1**).

**Table 1:** The association of TCS exposure concentration and the hatching rate of zebrafish embryos within 48 and 72 h.

Treatment	Fertilized eggs	Number of larvae hatched	
	n	Up to 48 hpf	Up to 72 hpf
TCS exposure levels (ng/mL)		n (%)	n (%)
0 (control)	600	252 (42.0%)	575 (95.8%)
3	600	287 (47.8%)	580 (96.7%)
30	600	207 (34.5%)	563 (93.8%)
100	600	141 (23.5%)	561 (93.5%)
300	600	169 (28.2%)	521 (86.8%)
900	600	26 (4.3%)	110 (18.3%)
*P*		<0.01*	<0.01*

Chi-square^*^ test was used to test the difference; hpf, hours post fertilization. Bold values mean statistical significant.

### TCS Exposure and Mortality of Zebrafish Larvae

All the larvae died shortly after hatching in 900 ng/mL TCS exposure group, and the mortality reached 100 % at 3 dpf. But in the TCS exposure 3 to 300 ng/mL groups, the mortalities of larvae were comparable to the control group at 7 dpf and 14 dpf, respectively (**Table 2**).

**Table 2:** The influence of TCS exposure on the larvae mortality in 14 days post fertilization.

Treatment	Larvae hatched	Larvae death	Number of larvae alive	Larvae death
	within 3 dpf	within 7 dpf	at the beginning of 8 dpf^#^	during 8 to 14 dpf
TCS exposure levels (ng/mL)	n	n ( %)	n	n ( %)
0 (control)	575	36 (6.3%)	389	16 (4.1%)
3	580	41 (7.1%)	389	18 (4.6%)
30	563	50 (8.9%)	363	18 (5.0%)
100	561	72 (12.8%)	339	9 (2.7%)
300	521	45 (8.6%)	326	9 (2.8%)
900	110	110 (100%)	——	——
*P*		< 0.01*		0.16

dpf, days post fertilization.

* Chi-square test was used.

^#^ number of larvae at the beginning of 8 dpf = total hatched larvae in 3 dpf number of larvae death up to 7 dpf – 150 larvae used for measuring thyroid hormones at the 7th day (i.e., 50 larvae/measure × 3 measures = 150 larvae for each exposure group). Bold values mean statistical significant.

### TCS Exposure and Thyroid Hormone Levels in Larvae and Zebrafish

We further measured the thyroid hormone levels in larvae after exposure to different concentrations of TCS at different time points. At 7 dpf, the TT_3_ and FT_3_ levels were comparable as control in 0 to 300 ng/mL TCS exposure groups; while the TT_4_ and FT_4_ levels were reduced with TCS exposure doses from 0 to 300 ng/mL, but the difference was not statistically significant (**Supplementary Table S1**).

At 14 dpf, the TT_3_ and FT_3_ levels in larvae were significantly lower in all the TCS exposure groups when compared to the control group. TT_3_ levels were on average 0.20 to 0.21 ng/mg lower in 3-300 ng/mL TCS exposure groups than that of the control group (all *P* < 0.05, **Table 3**), and the FT_3_ levels were 9.49, 10.74, and 9.32 pg/mg lower in TCS 30, 100, and 300 ng/mL groups, respectively (*P* < 0.01 , **Table 3**). The FT_4_ level tended to decrease with the increase of TCS exposure doses (*P* trend < 0.05, **Table 3**); while TT_4_ levels had no statistically significant changes in 0-300 ng/mL TCS exposure groups (**Table 3**).

**Table 3:** The influence of TCS exposure on the thyroid hormone level of zebrafish larvae at 14 days post fertilization.

Treatment	TT_3_ (ng/mg)		TT_4_ (nmol/g)		FT_3_ (pg/mg)		FT_4_ (pg/mg)	
TCS exposure levels (ng/mL)	mean ± SD	β (95% CI)	mean ± SD	β (95% CI)	mean ± SD	β (95% CI)	mean ± SD	β (95 % CI)
0 (control)	0.39 ± 0.06	Reference	2.24 ± 0.38	Reference	28.53 ± 1.98	Reference	0.72 ± 0.20	Reference
3	0.19 ± 0.08	-0.199 (-0.347, -0.050)^*^	1.74 ± 0.67	-0.493 (-1.253, 0.267)	25.82 ± 0.24	-2.708 (-8.626, 3.210)	0.63 ± 0.20	-0.084 (-0.436, 0.268)
30	0.18 ± 0.11	-0.206 (-0.355, -0.058)^*^	1.84 ± 0.36	-0.396 (-1.156, 0.363)	19.04 ± 5.10	-9.495 (-15.413, -3.577)^**^	0.43 ± 0.20	-0.287 (-0.639, 0.065)
100	0.18 ± 0.08	-0.203 (-0.352, -0.054)^*^	2.05 ± 0.36	-0.188 (-0.948, 0.572)	17.79 ± 3.63	-10.745 (-16.663, -4.827)^**^	0.55 ± 0.22	-0.172 (-0.524, 0.180)
300	0.19 ± 0.08	-0.192 (-0.341, -0.044)^*^	1.61 ± 0.13	-0.629 (-1.389, 0.130)	19.21 ± 3.13	-9.326 (-15.244, -3.408)^**^	0.38 ± 0.14	-0.341 (-0.693, 0.012)
*P* for trend		0.04^*^		0.23		0.0013^**^		0.04^*^

100 larvae in each exposure group for one measurement (n=3, with 100 larvae per n). ^*^P < 0.05 ^**^P < 0.01. Bold values mean statistical significant.

**Figure 1:**
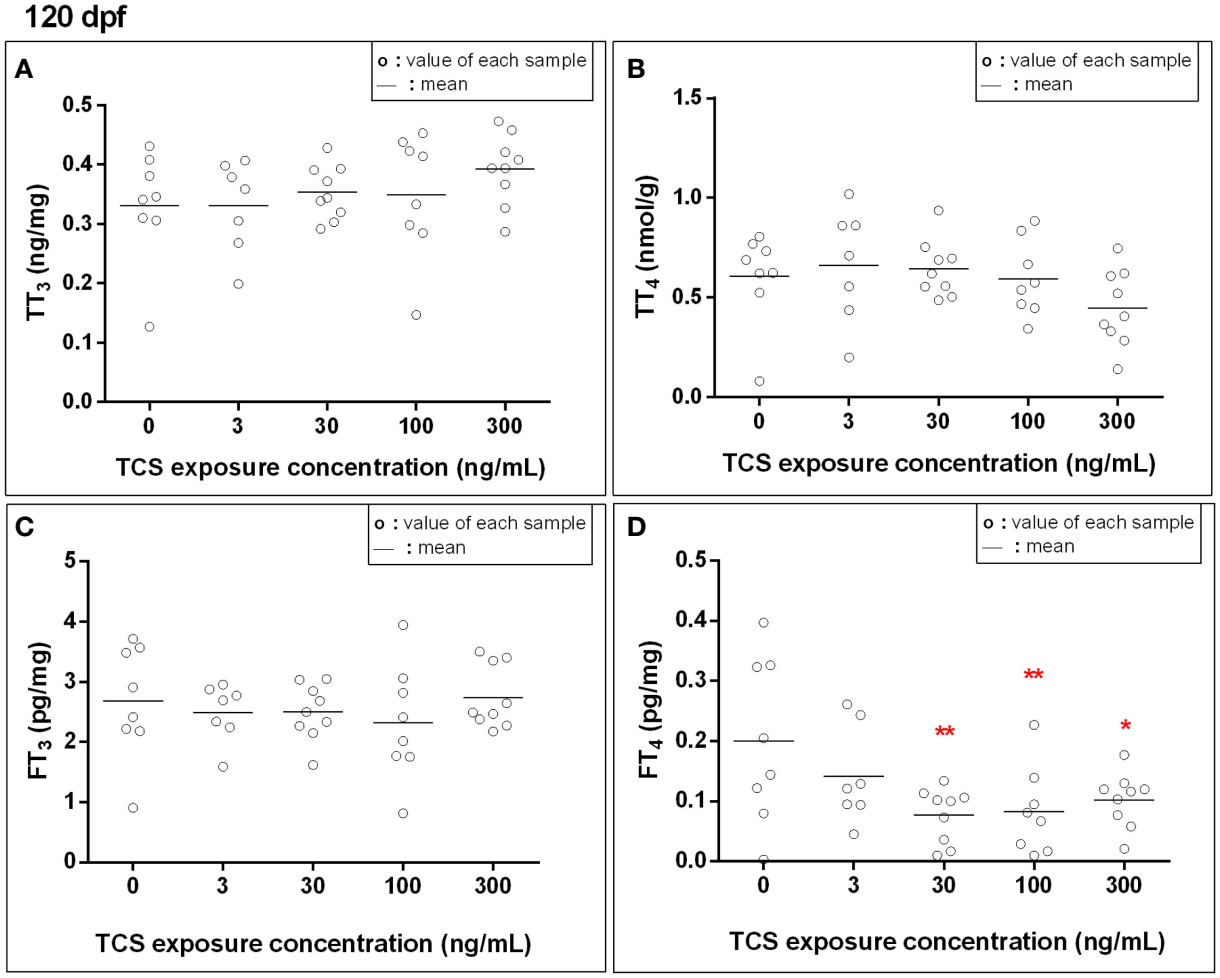
The thyroid hormone levels [**(A)**: total triiodothyronine, **(B)**: total thyroxine, **(C)**: free triiodothyronine, **(D)**: free thyroxine] in zebrafish exposed to TCS for 120 days after fertilizing. Thyroid hormone concentrations in fish at 120 dpf were measured for each exposure group, with each sample homogenized from every single fish at 120 dpf. *P* trend for FT_4_ = 0.01. *P < 0.05, **P < 0.01.

At 120 dpf, the FT_4_ levels were 0.14, 0.12, and 0.11 pg/mg lower in 30, 100, and 300 ng/mL TCS groups when compared to the control group, respectively (**Figure 1** and **Supplementary Table S2**, all *P* < 0.01). The TT_3_, TT_4_, and FT_3_ levels were comparable among the five TCS exposure groups. After adjusted for sex, the FT_4_ levels were still lower in TCS 30, 100, and 300 ng/mL groups than that in the control group (all *P* < 0.01, **Supplementary Table S3**).

### TCS Exposure and Thyroid Histopathological Change in Zebrafish

**Figure 2:**
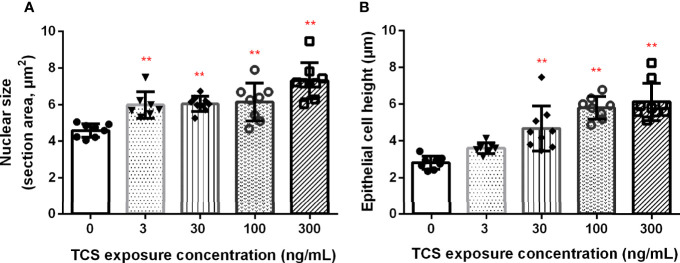
The association between TCS exposure concentrations and nuclear area **(A)** and the height **(B)** of thyroid follicle cells. *P* trend < 0.0001 both for nuclear size and epithelial height of the thyroid. ** *P*< 0.01 in the TCS exposure group vs. the control group.

**Figure 3:**
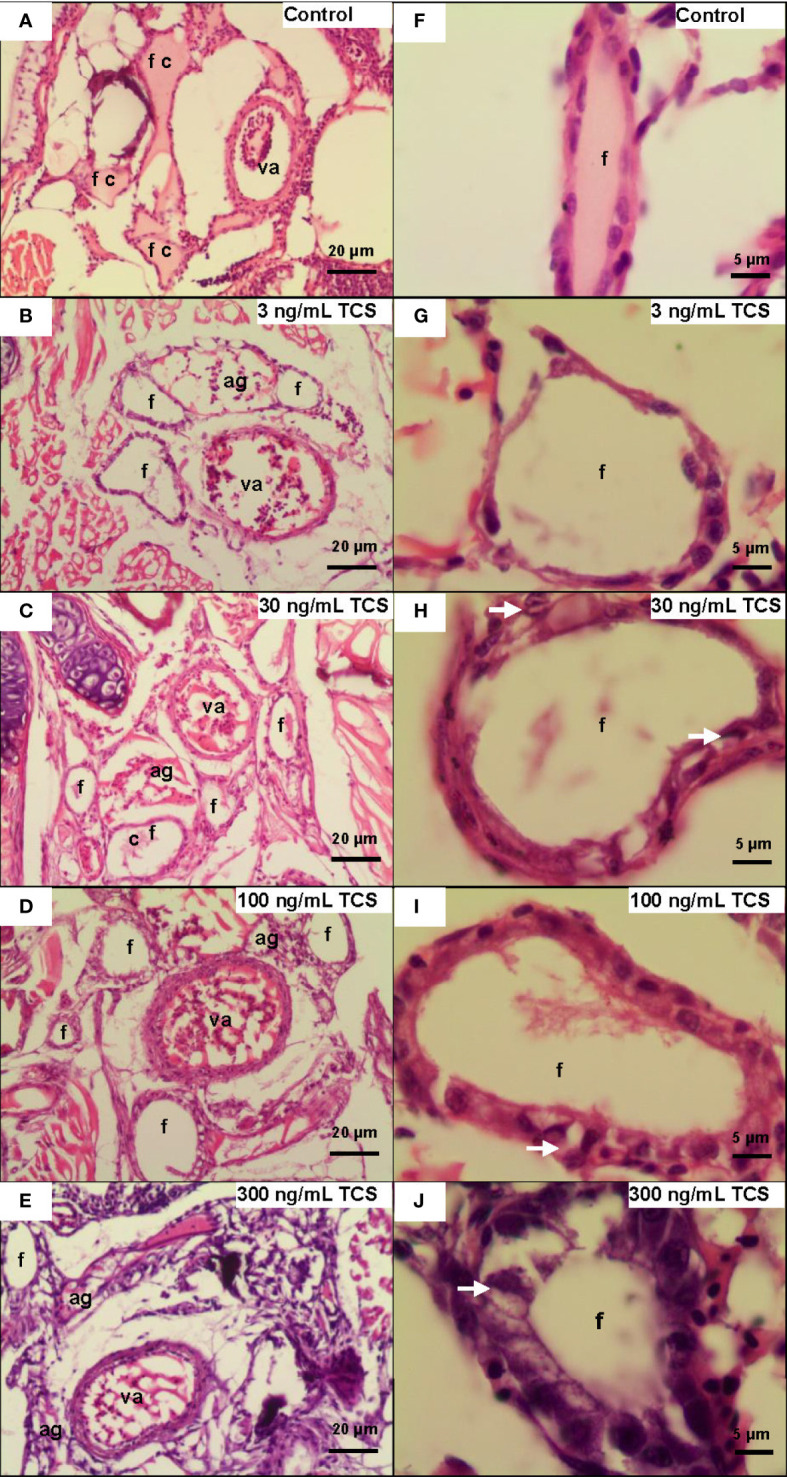
The morphological changes of thyroid follicles in different TCS concentration groups. **(A)** In the control group, the zebrafish follicle squamous to cuboidal follicular epithelium, with colloid in follicle at 400× and 1000× **(F)**. **(B–E)** Follicle hyperplasia with decreased colloid in the lumen and angiogenesis in TCS 3, 30, 100, and 300 ng/mL exposure at 400×. **(G–J)** Hypertrophy and hyperplasia of follicle cells in TCS 3, 30, 100, and 300 ng/mL exposure at 1000×. (va = ventral aorta; c = colloid; f = thyroid follicle; ag = angiogenesis; white arrows = hypertrophy).

We further examined the histological change of the thyroid gland in 120 dpf zebrafish. Ventral aorta was used to locate the dispersed thyroid follicles. In 3-300 ng/mL TCS exposure groups, the thyroid follicular epithelial cells became hyperplasia (increased number of follicular cell) and hypertrophy (enlarged follicular cell size and nuclear size), shown as increased nuclear area and cell height of thyroid follicle epithelial cells (**Figures 2** and **3**, **Supplementary Table S4**). At the same time, fish had oval thyroid follicles composed of a uniform monolayer of cuboidal thyrocytes and lumen filled with pink-stained colloid in most of the follicles in the control group (**Figures 3A, F**). But after exposure to TCS for 120 days, colloid depletion of follicles and angiogenesis (the development of new blood vessels from an existing vasculature) were observed (**Figures 3B–E** at 400×; **G, H, I**, and **J** at 1000×). TCS exposure induced follicle hyperplasia (**Figures 3C–E**), and nuclear hypertrophy ( **Figures 3G–J**), and the thickening of the layer of follicle epithelial cell in those follicles (**Figures 3C–E**). In the zebrafish exposed to high TCS levels at 100 ng/mL and 300 ng/mL, the thyroid follicle cells had obvious morphological alterations, for example, the follicle cells became significantly larger and follicular interstitial hyperplasia (**Figures 3C–E, G**).

## Discussion

In this study, we examined the disrupting effects of early exposure to TCS on the thyroid hormones in larvae and adult zebrafish, and the pathological changes of thyroid follicles. We found that the hatching rate of zebrafish embryos reduced whin 48 hpf with high TCS exposure, while the hatching rate became similar as the control when exposing to TCS at 0 - 300 ng/mL. TCS exposure did not change the mortality of larvae at 0 - 300 ng/mL, either. But in the extremely high TCS exposure (900 ng/mL), both the hatching rate and the survival of larvae were severely reduced. As to the effects of TCS on thyroid hormones, the levels of TT_3_ and FT_3_ were lower in larvae at 14 dpf in 30, 100, and 300 ng/mL TCS exposure, and the FT_4_ level was lower at 120 dpf in 30, 100, and 300 ng/mL TCS exposure. Moreover, with increasing concentration of TCS exposure, the epithelial height and nuclear size (area) of thyroid follicular cell turned larger in the thyroid gland of adult zebrafish at 120 dpf.

Zebrafish is an ideal animal model for study of chemical pollutants in water and thyroid hormone-disruption, partly due to its high (71 %) genetic similarity to humans (43). We set 2, 3, 7, and 14 days for larvae phase TCS exposure, 120 days (long-time) for adult phase TCS exposure (36). In this study, TCS exposure duration time was chosen according to the physiological development characteristics of zebrafish. The thyroid hormone level of zebrafish became stable after 7 dpf (44), and zebrafish reach sexual maturity at 120 dpf (45). Series of comparable TCS concentrations with natural and human exposure concentration were set in this study. Similar to our results, a previous study also found that significantly delayed hatchability of fertilized eggs and increased mortality of larvae in TCS 500 ng/mL exposure for 6 days (33). The influence of EDCs on zebrafish’s thyroid histopathology has been explored in many other related EDCs, such as perchlorate (46), arsenate (47), fluoride (48), and microcystin-LR (49). To date, there was only one study focused on the TCS exposure to adult zebrafish and thyroid histopathology (29). Consistent with our results, it reported that the thyroid follicle epithelium was changed in the sub-chronic (21 days) TCS treated fish (29). The reduction or increase in height of thyroid follicle cells are both the evidence of pathological diagnosis in thyroid dysfunction.

In previous studies, Schnitzler et al. (28) found that 20, 50, and 100 ng/mL TCS exposure to embryos decreased T_4_ and T_3_ levels at 9 days post-hatching (dph) and 12 dph, and increased T_4_ levels at 15 dph in *Cyprinodon variegatus*. Pinto et al. (29) assessed the effect and possible mechanism of TCS on adult zebrafish. They found that TCS exposure to adult zebrafish (about 100 μg/g per day for 21 days *via* diet) induced a reduction in circulating thyroid hormones, hyperplasia of follicles and the thyrocyte height. In our experiments, the TT_4_ and FT_4_ levels tended to be decreased with higher TCS exposure after 7 days, although they were not significant. The obvious thyroid disrupting effect by TCS shown at 14 dpf, and the FT_4_ decreased significantly with the increasing TCS exposure concentration both at 14 and 120 dpf. The impact of TCS exposure on thyroid hormone levels (TT_3_ and FT_3_) at 14 dpf provides the causal support for the findings in the human population (17). In the fish models, only three studies have examined the influence of TCS exposure on thyroid disruption (28, 29). Similar to our results, one study found that TCS exposure delayed the hatching of 6-13 h in medaka fish (28). A recent study found that the offspring of zebrafish exposed to TCS decreased the survival rate and delayed maturation (50), which might be partially explained by decreased thyroid hormone resulting from TCS exposure (50). The adverse effect of long-term exposure to TCS on thyroid hormone levels might be due to the cumulative effect of TCS on the development of zebrafish thyroid glands.

Most of the TT_4_ and a few of the TT_3_ are synthesized by thyroid follicular cells directly and then are transported to blood and be functioned in tissues (51). FT_3_ is the most active free form which comes from FT_4_ deiodination. FT_3_ and FT_4_, TT_3_ and TT_4_ can be transformed into each other and maintain dynamic balance in the blood (51). TT_4_ and TT_3_ are usually used as an indicator of the reserve capacity of the thyroid gland. In this study, under the high TCS exposure levels, TT_3_ and FT_3_, rather than TT_4_ or FT_4_, decreased statistically significantly in larvae at 14 dpf. Continuous exposing to 120 dpf, only the FT_4_ level decreased significantly. More studies about the mechanism still need to be explored.

In our study, since the larvae all died by day 3 after they were hatched, thyroid hormone levels were not measured in the 900 ng/mL TCS exposure group. We used the whole body of larvae fish at 7 and 14 dpf and the body without head, viscera, and gut of adult fish at 120 dpf to measure the thyroid hormone levels as most previous studies. In zebrafish, thyroid hormone is expressed in both hepatic and muscle (52). If adult zebrafish were used as models, plasma or body are generally used as biological samples (53). Thyroid hormone levels in muscle can reflect the growth and development of the body (52), and in our study, the thyroid hormone concentration in the muscle was found related to the TCS exposure level and therefore presumably affects the body growth of the zebrafish.

In this study, we selected the TCS exposure (3, 30, 100, 300, and 900 ng/mL) based on the environmental TCS levels, including wildlife and general population TCS exposure levels in a natural environment. The 3-100 ng/mL TCS is the range of urinary TCS concentrations in pregnant women (17), and 3-30 ng/mL is the TCS range in waste water (10). TCS was found in ranges from 5370 ng/L to 86,161 ng/L in wastewater treatment plants (10), and 1.85 ng/L to 9650 ng/L in rivers and surface water worldwide (9–11, 54, 55). The median urinary TCS concentration is 2.52 ng/mL (17, 19), while the highest urinary TCS concentrations in pregnant women was close to 100 ng/mL (17). It is about 25% of the lethal concentration (LC50) for zebrafish (33). In zebrafish, LC50 values of TCS were 420 μg/L (95% CI, 380 - 450 ng/mL) for embryos at 96 hpf, and 340 μg/L for adult zebrafish within 96 h (33).

The toxic effects of TCS were well recognized on bacterial resistance, reproductive toxicity, and cytotoxicity in aquatic organisms (56–58), but the results on thyroid hormone disruption were inconsistent (59, 60). Most of the animal studies mainly focused on rodents (24, 61). In the adult rat, TCS treatment reduced the levels of blood TT_4_ and FT_4_, but no change was found in the levels of blood thyroid stimulating hormone (TSH) (25, 62–64). Another study found that maternal TCS (300 mg/kg/day) exposure during pregnancy decreased approximately 30% of TT_4_ in dams at the postpartum day 22 in mice (64). Previous studies found that TCS exposure decreased thyroid hormone levels and inhibited metamorphosis (59, 65), or neither changed the thyroid hormone level nor altered the metamorphosis in *Xenopus laevis* (60).

We speculate that TCS may inhibit thyroid hormones secretion by acting as a disruptor of the hypothalamic-pituitary-thyroid (HPT) axis or due to the increasing thyroid hormones clearance. Some mechanisms have been studied but the data were controversial. TCS exposure may promote (29) or inhibit (66) the sodium-iodide symporter (NIS)-mediated iodide uptake in animals’ thyroid, then increase or decrease the thyroid hormone levels. TCS decreased the thyroid hormones in the circulation of zebrafish but induced TSH gene transcription by a negative feedback regulation (29). TCS was reported to inhibit the activity of thyroid peroxidase (TPO) and thyroid hormone synthesis at concentrations of 50 ng/mL in rat (66) , and increased T_4_ metabolism and decreased T_4_ bioavailability by increasing the hepatic enzymes activity of glucuronyltransferase and pentoxyresorufin-O-deethylase (PROD) in the liver (67–69), or by activating the nuclear receptor, pregnane X receptor (PXR) (24, 61). It was also found that TCS caused hypothyroidism in rats through p38/TRHr-dependent pathway (57). Therefore, the mechanism about TCS inhibiting the thyroid hormones is still unclear and needs further investigation.

The result in larvae of this study is consistent with our previous study in humans, in which we observed an inverse association between maternal urinary TCS and cord blood FT_3_ level in Chinese newborns (17). It is established that thyroid hormone is critical for intrauterine neurodevelopment, due to the regulation of migration, proliferation, and differentiation of fetal neuronal cells, as well as synaptogenesis and myelination (1, 2). The nervous system is highly thyroid hormone sensitive in prenatal life, especially in the early weeks of embryonic development (70).

Our study found that zebrafish exposed with TCS for 120 days (from embryo to adult phase) caused thyroid histopathology changes in 3, 30, 100, and 300 ng/mL TCS exposure, and disturbed the thyroid hormone level (FT_4_) in 30, 100, and 300 ng/mL TCS exposure. This is the first study to explore the thyroid disturbed effect by long-term TCS exposure starting from the early life in zebrafish. It has been reported that the formation, growth, and differentiation of thyroid follicles in zebrafish appear to be independent of TSH (71), although the TSH receptor is responsible for thyroid gland differentiation in zebrafish (72). Future study with measurements on TSH level can lead to exploring the potential disruptive effect of TCS on the feedback mechanism of HPT axis. Considering an environmental TCS exposure, we set various TCS concentrations in water from very low to high levels in our experiment but did not measure the direct TCS concentration in the fish.

We found that exposure to TCS delayed the hatching of zebrafish embryos, decreased the thyroid hormone (FT_3_ and TT_3_) levels in the zebrafish larvae and the FT_4_ level in adult zebrafish (even adjusted for age). Moreover, TCS exposure affected the structure of thyroid follicles by increasing nuclear area and epithelial cell height of zebrafish thyroid follicles. Considering the reduced level of thyroid hormones which are crucial for neurodevelopment (73), our study raises the concern that early TCS exposure (from conception) may profoundly influence child neurobehavioral development at such a highly sensitive window.

## Data Availability Statement

The raw data supporting the conclusions of this article will be made available by the authors, without undue reservation.

## Ethics Statement

The studies involving animals were reviewed and approved by the Ethics Committee of Xinhua Hospital Affiliated to Shanghai Jiao Tong University School of Medicine.

## Author Contributions

FO conceptualized the study. FO, NT, and PF implemented the study, FO and NT interpretated data and drafted the manuscript. FO, NT, PF, WYW, WJW, and XY contributed to acquisition and analysis of research data. LC had intensively revised the manuscript. All authors have reviewed and approved the manuscript as submitted.

## Funding

This study was supported by grants from the National Natural Science Foundation of China [grant number No. 81961128023; 81673178], Shanghai Municipal Education Commission - Gaofeng Clinical Medicine Grant [grant number 20152518], and in part by the Collaborative Innovation Program of Shanghai Municipal Health Commission [grant number 2020CXJQ01].

## Conflict of Interest

The authors declare that the research was conducted in the absence of any commercial or financial relationships that could be construed as a potential conflict of interest.

## Publisher’s Note

All claims expressed in this article are solely those of the authors and do not necessarily represent those of their affiliated organizations, or those of the publisher, the editors and the reviewers. Any product that may be evaluated in this article, or claim that may be made by its manufacturer, is not guaranteed or endorsed by the publisher.
