# Supplementary material for: The Effect of Early Life Exposure to Triclosan on Thyroid Follicles and Hormone Levels in Zebrafish
Source: Front Endocrinol (Lausanne). 2022 Jun 3;13:850231. doi: 10.3389/fendo.2022.850231 (PMC9203717; doi:10.3389/fendo.2022.850231)
Supplement: Supplementary file 1 [file Table_1.docx]

**Supplemental tables**

**Table S1. The influence of TCS exposure on the thyroid hormone level of zebrafish larvae at 7 days post fertilization**

| treatment | TT_3_ (ng/mg) | |  | TT_4_ (nmol/g) | |  | FT_3_ (pg/mg) | |  | FT_4_ (pg/mg) | |
| --- | --- | --- | --- | --- | --- | --- | --- | --- | --- | --- | --- |
| TCS exposure levels (ng/mL) | mean±SD | β (95% CI) |  | mean±SD | β (95% CI) |  | mean±SD | β (95% CI) |  | mean±SD | β (95% CI) |
| 0 (control) | 0.66±0.19 | Reference |  | 6.02±3.32 | Reference |  | 22.67±3.45 | Reference |  | 7.94±6.81 | Reference |
| 3 | 1.10±0.91 | 0.436(-0.858, 1.730) |  | 5.47±4.13 | -0.547(-6.477, 5.383) |  | 18.13±0.20 | -4.546(-9.779, 0.686) |  | 9.51±7.69 | 1.571(-8.946, 12.089) |
| 30 | 0.46±0.18 | -0.196(-1.490,1.098) |  | 5.60±4.93 | -0.425(-6.355, 5.505) |  | 17.46±4.81 | -5.210(-10.442, 0.023) |  | 6.78±6.43 | -1.163(-11.680, 9.354) |
| 100 | 1.32±0.88 | 0.660(-0.635, 1.954) |  | 3.17±0.33 | -2.855(-8.785, 3.074) |  | 18.17±2.08 | -4.508(-9.741, 0.724) |  | 5.04±4.48 | -2.902(-13.419, 7.615) |
| 300 | 1.23±0.93 | 0.575(-0.719, 1.870) |  | 2.30±0.74 | -3.718(-9.648, 2.212) |  | 18.69±1.38 | -3.981(-9.214, 1.251) |  | 0.30±0.28 | -7.644(-18.162, 2.873) |
| *P* for trend |  | 0.29 |  |  | 0.09 |  |  | 0.17 |  |  | 0.06 |

50 larvae in each exposure group for one measurement (n=3, with 50 larvae per n) ^*^*P* < 0.05

| treatment |  | TT_3_ (ng/mg) | |  | TT_4_ (nmol/g) | |  | FT_3_ (pg/mg) | |  | FT_4_ (pg/mg) | |
| --- | --- | --- | --- | --- | --- | --- | --- | --- | --- | --- | --- | --- |
| TCS exposure levels (ng/mL) | n | mean±SD | β (95% CI) |  | mean±SD | β (95% CI) |  | mean±SD | β (95% CI) |  | mean±SD | β (95% CI) |
| 0 (control) | 8 | 0.33±0.09 | Reference |  | 0.53± 0.22 | Reference |  | 2.68± 0.94 | Reference |  | 0.20± 0.14 | Reference |
| 3 | 7 | 0.33± 0.08 | -0.0003 (-0.082, 0.081) |  | 0.66± 0.28 | 0.133 (-0.081, 0.346) |  | 2.50± 0.48 | -0.179 (-0.921, 0.562) |  | 0.13± 0.09 | -0.073 (-0.159, 0.013) |
| 30 | 9 | 0.35± 0.05 | 0.022 (-0.054, 0.099) |  | 0.62± 0.14 | 0.089 (-0.111, 0.289) |  | 2.50 ± 0.46 | -0.177 (-0.874, 0.519) |  | 0.06± 0.04 | -0.134 (-0.218, -0.056)^**^ |
| 100 | 8 | 0.35± 0.10 | 0.018 (-0.061, 0.097) |  | 0.54± 0.18 | 0.013 (-0.192, 0.219) |  | 2.32± 0.96 | -0.353 (-1.069, 0.364) |  | 0.08± 0.07 | -0.123 (-0.206, -0.039)^**^ |
| 300 | 9 | 0.39± 0.06 | 0.061 (-0.016, 0.138) |  | 0.45± 0.19 | -0.084 (-0.284, 0.116) |  | 2.74± 0.53 | 0.068 (-0.629, 0.764) |  | 0.09± 0.04 | -0.109 (-0.190, -0.028)^**^ |
| *P* for trend |  |  | 0.66 |  |  | 0.06 |  |  | 0.42 |  |  | 0.07 |

**Table S2.** **The influence of TCS exposure on the thyroid hormone level of zebrafish at 120 days post fertilization**

TT_4_, FT_4_ below the limit of detection (LOD) were replaced with LOD/2. (LOD of TT_3_ = 0.1 ng/mL; LOD of TT_4_ = 8 nmol/L; LOD of FT_3_ = 0.3 pg/mL; LOD of FT_4_ = 1 pg/mL)

^*^*P* < 0.05 ^**^*P* <0.01

| treatment |  | TT_3_ (ng/mg) | | TT_4_ (nmol/g) | |  | FT_3_ (pg/mg) | |  | FT_4_ (pg/mg) | |
| --- | --- | --- | --- | --- | --- | --- | --- | --- | --- | --- | --- |
| TCS exposure levels (ng/mL) | n | mean±SD | β (95% CI) | mean±SD | β (95% CI) |  | mean±SD | β (95% CI) |  | mean±SD | β (95% CI) |
| 0 (control) | 8 | 0.33±0.09 | Reference | 0.53± 0.22 | Reference |  | 2.68± 0.94 | Reference |  | 0.20± 0.14 | Reference |
| 3 | 7 | 0.33± 0.08 | -0.017 (-0.088, 0.053) | 0.66± 0.28 | 0.099 (-0.101, 0.299) |  | 2.50± 0.48 | -0.341 (-0.971, 0.289) |  | 0.13± 0.09 | -0.076 (-0.164, 0.012) |
| 30 | 9 | 0.35± 0.05 | 0.018 (-0.048, 0.084) | 0.62± 0.14 | 0.080 (-0.106, 0.266) |  | 2.50 ± 0.46 | -0.219 (-0.806, 0.367) |  | 0.06± 0.04 | -0.138 (-0.220, -0.056)^**^ |
| 100 | 8 | 0.35± 0.10 | 0.018 (-0.050, 0.085) | 0.54± 0.18 | 0.013 (-0.178, 0.205) |  | 2.32± 0.96 | -0.352 (-0.956, 0.251) |  | 0.08± 0.07 | -0.123 (-0.207, -0.039)^**^ |
| 300 | 9 | 0.39± 0.06 | 0.057 (-0.009, 0.122) | 0.45± 0.19 | -0.093 (-0.279, 0.094) |  | 2.74± 0.53 | 0.026 (-0.561, 0.613) |  | 0.09± 0.04 | -0.110 (-0.192, -0.028)^**^ |
| *P* for trend |  |  | 0.36 |  | 0.08 |  |  | 0.16 |  |  | 0.07 |

**Table S3. The influence of TCS exposure on the thyroid hormone level of zebrafish at 120 days post fertilization adjusted for sex**

TT_4_, FT_4_ below the limit of detection (LOD) were replaced with LOD/2. (LOD of TT_3_ = 0.1 ng/mL; LOD of TT_4_ = 8 nmol/L; LOD of FT_3_ = 0.3 pg/mL; LOD of FT_4_ = 1 pg/mL)

^*^*P* < 0.05 ^**^*P* <0.01

**Table S4. Effects of TCS exposure on nuclear area and height of thyroid follicular cells**

|  |  | area of nuclear (μm^2^) | | |  | height of follicular epithelial cell (μm) | | |
| --- | --- | --- | --- | --- | --- | --- | --- | --- |
| TCS concentration | n | mean±SD | β (95% CI) | *P*-value |  | mean±SD | β (95% CI) | *P*-value |
| 0 (control) | 8 | 4.59±0.37 | Reference |  |  | 2.82±0.35 | Reference |  |
| 3 ng/ml | 7 | 5.98±0.73 | 1.399 (0.603, 2.194) | 0.0011 |  | 3.60±0.30 | 0.783 (-0.080, 1.646) | 0.074 |
| 30 ng/ml | 9 | 6.05±0.41 | 1.461 (0.714, 2.208) | 0.0003 |  | 4.68±1.22 | 1.861 (1.050, 2.671) | <0.0001 |
| 100 ng/ml | 8 | 6.15±1.04 | 1.560 (0.791, 2.329) | 0.0002 |  | 5.79±0.61 | 2.975 (2.141, 3.809) | <0.0001 |
| 300 ng/ml | 9 | 7.31±0.98 | 2.723 (1.978, 3.472) | <0.0001 |  | 6.11±1.02 | 3.291 (2.480, 4.101) | <0.0001 |
| *P* |  |  | <0.0001 |  |  |  | <0.0001 |  |
